# Melbourne Notes

**Published:** 1909-08-21

**Authors:** 


### Melbourne Notes.

(From a Correspondent.)

An impression is gaining ground that our lunacy administration is not all that it should be, at least our percentage of recoveries appears to be the lowest but one of all the States. Dr. Jones, Inspector-General of the Insane, accounts for this "appearance" by the large number of discharges from the Receiving House—opened about three years ago—that would formerly have been discharged from the Asylums, and also by our use of the "trial leave," the patients on "trial leave" being recorded as "relieved" rather than as "recovered." Whatever the reason of the fall of recoveries from 44.97 per cent. in 1902 to 30.08 per cent. in 1907, the uneasy public mind is demanding investigation. Dr. Norris, too, lately chairman of the Board of Public Health, has been making a stir about mental cases in private hospitals not licensed for the insane, and where the conditions necessary for mental recovery are absent. Further investigation and legislation are promised.

By the Constitution of the Commonwealth of Australia quarantine was made a Federal Department, but only now has Parliament assumed control, and the Federal Quarantine Act came into operation on July 1. With Eastern ports within a few days' sail, and smallpox and bubonic plague for ever threatening our 5,000 miles of coast, quarantine—human, animal, and vegetable—is a matter of deadly earnest to Australia. Hitherto it has been left to each State to conduct its own quarantine, which has been woefully lax in all. The Department is yet to be created practically, and the Director will be busy for a long time in framing regulations especially for the safeguarding of the three "gates"—Freemantle, Port Darwin, and Thursday Island. Dr. W. P. Norris, the Director of the new Federal Department, was for nearly five years medical inspector and chairman of the Board of Public Health, in succession to the late regretted Dr. D. Astley Greswell, under whom Dr. Norris was senior assistant medical inspector. The present senior assistant is Dr. Robertson, a native-born Victorian, who solely by his own energy and determination has passed through the Universities of Melbourne, Edinburgh, and Cambridge, securing the diploma of public health at the last-named, and the Fellowship of the Royal College of Surgeons. He was appointed second assistant medical inspector in 1901, and first in 1904. Now he is likely to succeed Dr. Norris.

Our Government certainly looks well after its citizens in their material welfare at least. At a sacrifice of revenue it prohibited the importation of opium; pure food and unwatered milk is its constant care. Within the last month it has undertaken to provide its primary school children with "physical culture," and has commenced a campaign for the eradication of consumption, one feature of which is the denial of hotel accommodation to persons suffering from phthisis. Fear of this disease is widespread. The Home for Consumptives at Echuca, a small town on the Murray, had to be abolished several years ago in deference to the long-continued protests of the townspeople. Now a newspaper discussion has been started by a few lay and medical men under the heading "National Defence against Disease," the idea being to nationalise medicine, so that the army of medical men and women as civil servants, paid by the State, would be free not only to heal, but without fear to teach the laws of health, the right way of living, and give equal medical attention to all. It would appear that the majority already do these things without "nationalising." The subject has not yet passed beyond the sphere of newspaper discussion.

